# Personalized Nutrition Intervention Improves Health Status in Overweight/Obese Chinese Adults: A Randomized Controlled Trial

**DOI:** 10.3389/fnut.2022.919882

**Published:** 2022-06-22

**Authors:** Juntao Kan, Jiayi Ni, Kun Xue, Feijie Wang, Jianheng Zheng, Junrui Cheng, Peiying Wu, Matthew K. Runyon, Hongwei Guo, Jun Du

**Affiliations:** ^1^Nutrilite Health Institute, Shanghai, China; ^2^Research Institute of the McGill University Health Center, Montreal, QC, Canada; ^3^School of Public Health, Fudan University, Shanghai, China; ^4^Department of Nutrition, Shanghai General Hospital, Shanghai, China; ^5^Nutrilite Health Institute, Ada, MI, United States

**Keywords:** personalized nutrition, diet, physical activity, genotype, phenotype, clinical trial

## Abstract

**Background:**

Overweight and obesity increase the risk of noncommunicable diseases (NCDs). Personalized nutrition (PN) approaches may provide tailored nutritional advice/service by focusing on individual's unique characteristics to prevent against NCDs.

**Objective:**

We aimed to compare the effect of PN intervention with the traditional “one size fits all” intervention on health status in overweight/obese Chinese adults.

**Methods:**

In this 12-week randomized controlled trial, 400 adults with BMI ≥24 kg/m^2^ were randomized to control group (CG, *n* = 200) and PN group (PNG, *n* = 200). The CG received conventional health guidance according to the *Dietary Guidelines for Chinese Residents* and *Chinese DRIs Handbook*, whereas the PNG experienced PN intervention that was developed by using decision trees based on the subjects' anthropometric measurements, blood samples (phenotype), buccal cells (genotype), and dietary and physical activity (PA) assessments (baseline and updated).

**Results:**

Compared with the conventional intervention, PN intervention significantly improved clinical outcomes of anthropometric (e.g., body mass index (BMI), body fat percentage, waist circumference) and blood biomarkers (e.g., blood lipids, uric acid, homocysteine). The improvement in clinical outcomes was achieved through behavior change in diet and PA. The subjects in the PNG had higher China dietary guidelines index values and PA levels. Personalized recommendations of “lose weight,” “increase fiber” and “take multivitamin/mineral supplements” were the major contributors to the decrease of BMI and improvement of lipid profile.

**Conclusion:**

We provided the first evidence that PN intervention was more beneficial than conventional nutrition intervention to improve health status in overweight/obese Chinese adults. This study provides a model of framework for developing personalized advice in Chinese population.

Chictr.org.cn (ChiCTR1900026226).

## Introduction

Overweight and obesity increase the risk of non-communicable diseases (NCDs), including diabetes, cardiovascular diseases, non-alcohol fatty liver disease, and even certain cancers (1, 2). The rising trends in overweight and obesity have plateaued in many high-income countries, but have accelerated in most Asian countries (1). Effective prevention strategies are required to reduce the immense global burden of nutrition-related NCDs, especially those primarily related to poor lifestyle choices, including unhealthy dietary patterns and insufficient physical activity (PA) (3, 4). Most population strategies to reduce the burden of NCDs have used conventional ‘one size fits all' public health recommendations throughout the past decades, but with limited efficacy (5).

Personalized nutrition (PN) approaches, which provide nutrition advice, products, and services tailored for an individual according to his/her unique biological characteristics, may provide extra motivations to the individual, and therefore, be more efficacious in preventing against NCDs (6). Zeevi et al. created personalized diets using a machine learning algorithm that integrated parameters including dietary habits, PA, and gut microbiota, to successfully lower postprandial blood glucose and its metabolic consequences (7). Mathers et al. carried out the Food4Me randomized controlled trial (RCT) in seven European countries and showed that decision tree-based PN intervention produced larger and more appropriate changes in dietary behavior compared with the conventional approaches. These studies collectively triggered enthusiasm on PN research in recent years (5, 8).

PN at the individual level requires not only the comprehensive collection of information, including anthropometric data, dietary intake, PA, blood biomarkers, and genotypes—which is both costly and demanding—but also models that are capable of accurately generating personalized advice for each individual (9, 10). Food4Me decision trees have been validated in several PN trials (5, 8, 11, 12). In the current study, the principal investigator and registered dietitians certificated by the China Nutrition Society revised the decision trees and accompanying advice messages to keep them in accordance with the Chinese dietary habits and technical standards. Using the localized decision trees, along with the PN report prepared by the registered dietitians, we carried out the first RCT on PN in China (Diet2Me study) to investigate whether PN intervention would show greater benefits to health status in overweight/obese adults compared with the conventional intervention.

## Methods

### Study Design

**Figure 1:**
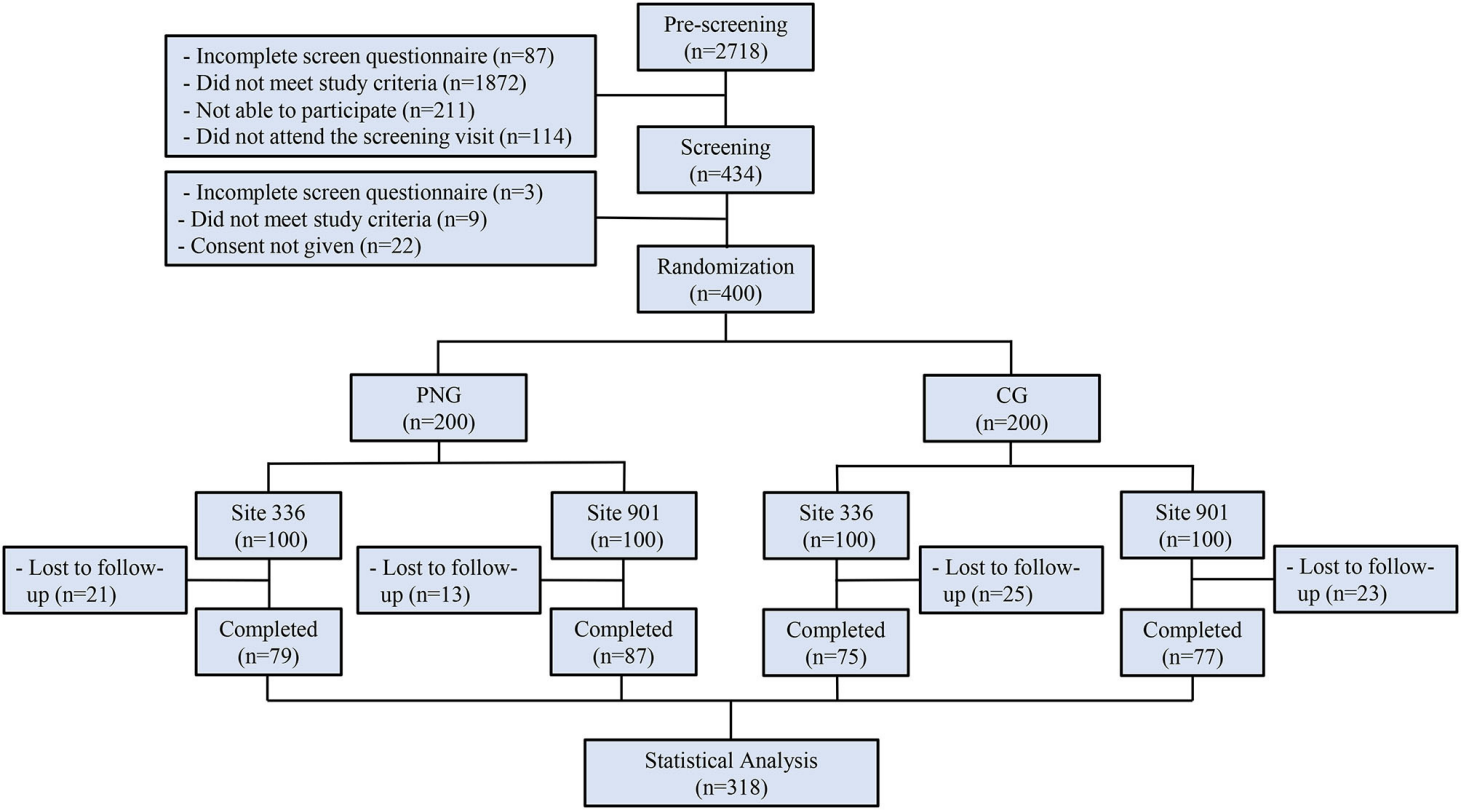
Flowchart of the clinical trial.

**Figure 2:**
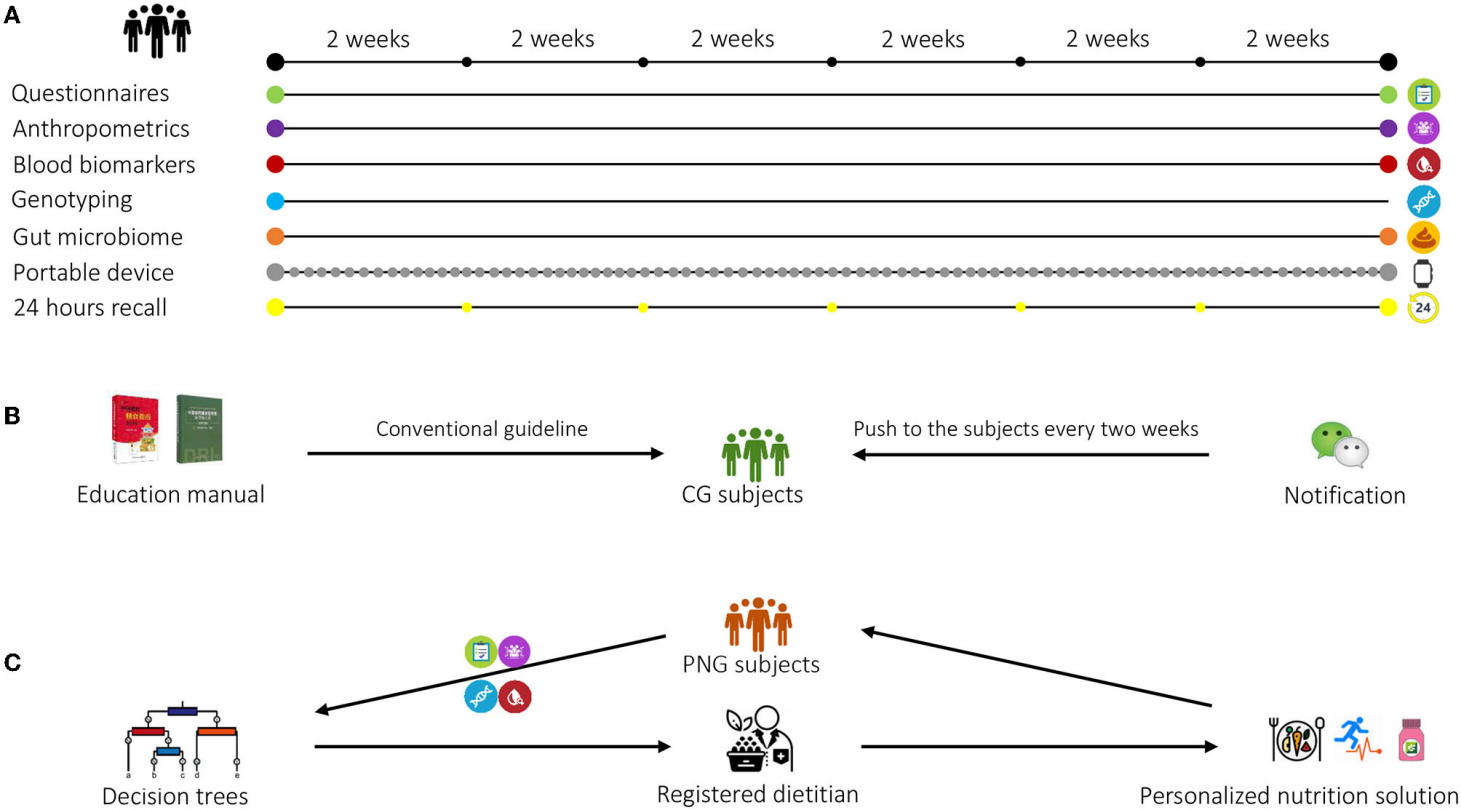
Overview of the study design. **(A)** Questionnaires, anthropometric measurements, blood, and fecal samples were collected at baseline and at end point. Buccal cells were collected at baseline for genotyping. During the intervention, 24-h recall was collected every 2 weeks, and a portable device was worn every day. **(B)** The subjects in the control group (CG) received non-personalized, conventional health guidance on diet, physical activity (PA), and nutritional supplements intake based on the *Dietary Guidelines for Chinese Residents* and *Chinese DRIs Handbook*. A brochure summarizing the health guidance was delivered to each CG subject at baseline, and follow-up notifications on nutrition education were provided biweekly to these subjects using WeChat. **(C)** The subjects in the personalized nutrition group (PNG) received personalized nutrition solution on diet, physical activity (PA), and nutritional supplements intake from the registered dietitians based on the decision trees. The personalized solution was updated biweekly based on the subjects' dietary and PA changes.

This randomized, controlled, multicenter clinical trial was conducted in Aier Hospital and Songnan Hospital, Shanghai, China. The participants were identified from the hospital databases. A total of 400 participants were initially enrolled in the study at baseline and randomized with a 1:1 ratio into two groups: control group (CG) and personalized nutrition group (PNG) (Figure 1). For both groups, questionnaires assessing diet and PA, anthropometric measurements, blood samples, buccal cells, and fecal samples were collected at baseline. During the intervention, 24-h dietary recall was collected every 2 weeks, and portable devices were worn every day. At the end point, the questionnaires, anthropometric measurements, blood, and fecal samples were collected again from both groups for analysis (Figure 2A). The primary outcome was body mass index (BMI). The secondary outcomes were other clinical outcomes of anthropometrics and blood biomarkers. No changes to trial outcomes after the trial commenced. Informed consent was obtained from all the patients. This trial was approved by the Institutional Review Board (IRB) of the Shanghai Nutrition Society and registered at chictr.org.cn (ChiCTR1900026226).

### Inclusion/Exclusion Criteria

The inclusion criteria encompassed either of the sexes, age 25 to 50 years, and overweight/obesity with BMI ≥24 kg/m^2^. Subjects were excluded if they had coronary heart disease, cerebrovascular disease, liver disease, kidney disease, inflammatory bowel disease, or hematologic disease; were pregnant or lactating women; were currently taking any drugs or dietary supplements in recent days, which could interfere with the study product; participated in another clinical trial in the past 3 months; or were not willing to comply with the study procedures.

### Intervention

The subjects in CG received a non-personalized, conventional health guidance on diet, PA, and nutritional supplements intake based on *Dietary Guidelines for Chinese Residents* and *Chinese DRIs Handbook* (Figure 2B). The guidelines embody the recommended proportion of the different food groups in the diet, recommendations to drink plenty of water, PA, dietary supplement intake, and dietary recommendations for specific population groups. A brochure summarizing the health guidance was delivered to each CG subject at baseline. Differed from the conventional nutrition intervention, a personalized solution integrating recommendations on diet, PA, and dietary supplements (Nutrilite, Guangzhou, China) was generated by registered dietitians based on decision trees, and was biweekly updated according to the dietary recall and PA assessments during the study (Figure 2C). The interventions to the CG and PNG were delivered through sending notifications via a localized messaging and calling application (WeChat) with the same frequency (biweekly) to ensure that both groups underwent the same intensity of intervention throughout the study. Neither CG nor PNG had actual interactions with dieticians to minimize bias between the groups. More details on the personalized intervention were described in the following sections. The list of dietary supplements is shown in Supplementary Table 1.

### Decision Trees

The decision trees used herein originated from the Food4Me study decision trees, which were kindly provided by Prof. John C. Mathers (5, 8). Considering the differences in dietary habits and technical standards between Western and Eastern countries, the decision trees and corresponding feedback messages were revised by Prof. Hongwei Guo, the principal investigator of the current study, in addition to five other registered dietitians certificated by the Chinese Nutrition Society. There were 16 decision trees in the current study, covering the majority of macro- and micronutrients.

### Personalized Nutrition Report

The PN report included a summary of the individual's anthropometric, nutrient, blood, and genetic (nutrition-related) profiles, an overall goal established by the registered dietitian, as well as personalized recommendations on lifestyle modifications. All the PN reports were prepared manually by registered dietitians using the revised decision trees. In brief, the numerical data collected at baseline (anthropometric characteristics, blood biomarkers, food intake, PA) were categorized into three levels: green (normal), yellow (higher or lower in moderation), and red (severely higher or lower), based on the Chinese standards published by the Chinese Nutrition Society and Chinese Medical Association (13, 14). Then, the categorized parameters were ranked, and 3–5 targeted goals were identified by registered dietitians. Specific messages related to each goal were developed according to the decision trees to advise changes in dietary habits and PA (5, 15). A nutritional supplement was suggested when the calculated intake of a certain nutrient was lower than 80% of the estimated average requirement (EAR). The complete report was delivered to each subject at baseline. In the follow-up period, the subjects received updated report sent by the dietitian every 2 weeks. This updated report was prepared based on the most recently collected 24 h-recall and wearable device recording, and just included the main goals and some follow-up comments on the behavior changes regarding to the individual goal.

### Anthropometric Measurements

Body weight (BW) and body height (BH) were measured using a standardized digital weight scale (Seca, Hamburg, Germany) and height meter (Seca). BMI is defined as weight in kilograms divided by the square of height in meters (kg/m^2^). Waist circumference (WC) and hip circumference (HC) was measured using a non-stretch tape, and waist-to-hip ratio (WHR) was calculated accordingly. Body composition was assessed utilizing an InBody (Biospace Co., Ltd., Seoul, Korea), in which body fat percentage (BFP) was recorded. Systolic blood pressure (SBP) and diastolic blood pressure (DBP) were measured by an electronic sphygmomanometer (Omron, Kyoto, Japan). All measures were taken twice at least. A third measure was taken when substantial discrepancies (difference ≥ 1%) were observed in the first two attempts.

### PA Assessment

Intensity (vigorous, moderate, or walking), frequency, and duration of PA were recorded using the short version of the International Physical Activity Questionnaire (IPAQ) to measure the PA level (16). The minutes/week of each PA intensity was calculated. According to the IPAQ scoring protocol, the metabolic equivalents of task (METs)-min/week were obtained by multiplying the average energy expenditure by min/week for each PA intensity (8.0 MET for vigorous intensity, 4.0 MET for moderate intensity, and 3.3 MET for walking) (17). Total PA score in MET-min/week was obtained by summing the results of each intensity of PA. The continuous scores were then categorized into three levels (low, moderate, and high) of PA to classify populations. In addition, step count, consumed energy, and sleep condition were recorded by Mi Band (Xiaomi, Beijing, China), a wearable device, in real time.

### Diet Assessment

A semiquantitative food frequency questionnaire (FFQ) approved by the Chinese Center for Disease Control and Prevention was slightly revised in the current study to assess the dietary intake of the subjects (18). The questionnaire contained 77 food items grouped into 13 categories: staple food, bean, vegetable, fungi/algae, fruit, dairy, meat, fish, egg, snack, drink, oil/spices, and smoke/alcohol. For each food item, the frequency and amount of consumption over the past week were recorded. China dietary guidelines index (CDGI) was calculated accordingly to evaluate the overall diet quality of the subjects. The details on the CDGI calculation were previously published (19).

The participants also used 24-h dietary recalls to record the name and amount of each food consumed for breakfast, lunch, dinner, and snacks. Food composition analysis was performed using commercially available software (Feihua, Beijing, China).

### Blood Biomarkers

Blood samples were collected after over 12-h fasting. The serum levels of triglycerides (TG), total cholesterol (TC), low-density lipoprotein (LDL), high-density lipoprotein (HDL), glucose (GLU), uric acid (UA), homocysteine (HCY), aspartate aminotransferase (AST), and alanine aminotransferase (ALT) were determined by colorimetry. The levels of vitamin A (VA), 25(OH)D_3_ (VD), docosahexaenoic acid (DHA), and eicosapentaenoic acid (EPA) were determined by tandem mass spectrometry. The levels of insulin (INS), vitamin B9 (folic acid, VB9) and vitamin B12 (VB12) were determined by chemiluminescence. The levels of calcium (Ca), magnesium (Mg), iron (Fe), zinc (Zn), and copper (Cu) were determined by atomic absorption spectrometry.

### Genotyping

Buccal cells were collected with oral swab (DNA Genotek, Ottawa, ON, Canada). DNA was extracted and analyzed by WuXi NextCode (Shanghai, China) using an Asian Single Nucleotide Polymorphism (SNP) Screening Chip (Illumina, San Diego, California, USA). The list of nine nutrition-related genes, including *BCO1, GC, MTHFR, FTO, APOE, FADS1, TCF7L2, CaSR*, and *TMPRSS6*, is shown in Supplementary Table 2. The gene variants were selected based on the documented evidence of gene-diet interactions (20–28). A systematic review of the rationale for selection of genes will be published separately soon.

### Sample Size Calculation

It was estimated that a total sample size of 286 participants (143 per group) would provide 80% power to detect a more than 5% change in BMI (29) between the two groups, with a significance level of 0.05. Allowing for a potential dropout rate of 25% during the intervention, we recruited 400 subjects (200 for each group) at baseline.

### Statistical Analysis

Statistical analyses were completed using SAS 9.4 (SAS Institute Inc., Cary, NC, USA). All statistical tests were two-sided and performed at the 0.05 significance level. Means and standard deviations (SD) were summarized for continuous variables with a normal distribution; medians and quartiles were provided for non-normally distributed variables; and frequencies and percentages were provided for categorical variables. Differences between the groups at baseline were assessed using analysis of variance (ANOVA) for continuous variables, and chi-square test for nominal categorical variables. Evaluations of the intervention effect were performed using analysis of covariance (ANCOVA) for normally distributed continuous variables, adjusted for baseline values. Log transformation was applied to non-normal continuous variables. Wilcoxon–Mann–Whitney test was used for highly skewed variables. The connections of longitudinal correlation network were calculated by Spearman or Point-Biserial and then plotted as a network by Cytoscape 3.8.2 (Cytoscape Consortium, San Diego, CA, USA).

## Results

### Study Design and Baseline Characteristics

A total of 2,718 participants were screened, of whom 400 eligible subjects were randomized into two groups: control group (CG) and personalized nutrition group (PNG). During 12-week intervention, 82 subjects withdrew, resulting in a total dropout rate of 20.5% (Figure 1). The subjects in PNG received a personalized nutrition solution on diet, PA, and nutritional supplements intake, which was provided by registered dietitians using decision trees. The subjects in CG received non-personalized, conventional health guidance (Figure 2). The baseline characteristics of the subjects, including demographics and anthropometrics, were comparable between the two groups (Table 1).

**Table 1:** Baseline characteristics of the subjects who completed the study.

	**CG** ***N* = 152**	**PNG** ***N* = 166**	***P*-value**
Male (%)	60 (39.5)	71 (42.8)	0.551
Age (years)	38.1 ± 7.5	38.8 ± 7.5	0.415
25–34	64 (42.1)	69 (41.6)	
35–50	88 (57.9)	97 (58.4)	
Education (%)			0.441
≤ 9 years	9 (5.9)	15 (9.0)	
9–12 years	15 (9.9)	20 (12.1)	
>12 years	128 (84.2)	131 (78.9)	
Family monthly income (%)			0.572
<20 000 RMB	16 (10.5)	24 (14.5)	
20,000–30,000 RMB	76 (50.0)	79 (47.6)	
≥30 000 RMB	60 (39.5)	63 (38.0)	
Weight (kg)	72.7 ± 10.6	73.8 ± 12.0	0.378
Height (cm)	163.1 ± 8.8	164.3 ± 9.1	0.237
BMI (kg/m^2^)	27.3 ± 3.4	27.2 ± 2.8	0.834
Body fat percentage (%)	27.2 ± 5.3	27.5 ± 5.1	0.646
Waist circumference (cm)	92.4 ± 9.9	92.6 ± 9.5	0.863
Waist-to-hip ratio	0.92 ± 0.08	0.92 ± 0.08	0.801
Systolic pressure (mm Hg)	125.1 ± 20.4	123.2 ± 17.4	0.368
Diastolic pressure (mm Hg)	84.2 ± 9.7	84.2 ± 11.2	0.995
Smoking (%)	21 (13.8)	18 (10.8)	0.420
Alcohol (%)	28 (18.4)	26 (15.7)	0.513

*Data are presented as frequency (%) or mean ± standard deviation (SD)*.

*Intergroup differences were evaluated using chi-square test or one-way analysis of variance (ANOVA)*.

*BMI, body mass index; CG, control group; PNG, personalized nutrition group*.

### Effect of PN Intervention on Anthropometric Measurements and Blood Biomarkers

**Figure 3:**
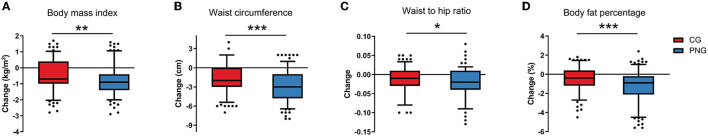
Changes in anthropometric measurements between CG and PNG. Intergroup differences in changes in anthropometric measurements **(A–D)** were evaluated using Wilcoxon–Mann–Whitney test. *P < 0.05, **P < 0.01, ***P < 0.001.

After 12 weeks of intervention, the subjects in both CG and PNG experienced significant improvement in weight, BMI, body fat percentage, waist circumference, and waist-to-hip ratio (Supplementary Table 3). The effects of PN intervention on decreasing BMI, body fat percentage, waist circumference, and waist-to-hip ratio were significantly more potent than those in CG (Figure 3). In addition, PN intervention was more efficient than the conventional intervention in decreasing blood levels of TG, TC, LDL, uric acid, and homocysteine (Table 2, Supplementary Table 4).

**Table 2:** Blood biomarkers at baseline and at week 12 in both groups.

		**CG** ***N* = 152**	**PNG** ***N* = 166**	**Between-group difference**	
				**LS Mean (95% CI)**	***P*-value**
TG, mmol/L ^a^	Baseline	1.12 (0.82, 1.68)	1.27 (0.93, 1.81)	−0.03 (−0.05, −0.001)	0.045
	Week 12	0.99 (0.76, 1.36)	1.03 (0.72, 1.41)		
TC, mmol/L	Baseline	5.21 (0.93)	5.28 (0.93)	−0.15 (−0.25, −0.05)	0.004
	Week 12	5.07 (0.88)	4.97 (0.89)		
HDL, mmol/L	Baseline	1.51 (0.28)	1.51 (0.26)	0.04 (−0.003, 0.08)	0.067
	Week 12	1.59 (0.31)	1.63 (0.28)		
LDL, mmol/L	Baseline	2.93 (0.71)	2.94 (0.72)	−0.10 (−0.19, −0.02)	0.020
	Week 12	2.67 (0.69)	2.58 (0.77)		
Glucose, mmol/L ^a^	Baseline	4.70 (4.45, 5.20)	4.70 (4.50, 5.30)	−0.005 (−0.02, 0.01)	0.455
	Week 12	4.50 (4.10, 5.25)	4.60 (4.30, 5.10)		
Insulin, μU/mL ^a^	Baseline	10.58 (6.24, 15.90)	8.98 (5.93, 12.34)	−0.04 (−0.10, 0.01)	0.143
	Week 12	9.39 (5.64, 15.33)	7.78 (5.59, 10.79)		
Uric acid, mmol/L	Baseline	328.07 (80.50)	337.08 (93.59)	−10.36 (−19.39, −1.33)	0.025
	Week 12	320.65 (74.85)	316.85 (76.49)		
ALT, U/L ^a^	Baseline	28.75 (17.45, 44.50)	25.65 (15.90, 40.70)	−0.04 (−0.08, 0.01)	0.150
	Week 12	28.10 (21.15, 57.05)	24.30 (17.00–40.80)		
AST, U/L ^a^	Baseline	23.60 (18.20, 31.35)	22.30 (17.90, 28.80)	−0.01 (−0.05, 0.02)	0.539
	Week 12	23.15 (18.90, 34.65)	21.75 (18.80–27.80)		
Vitamin A, mg/L	Baseline	1.04 (0.29)	1.01 (0.27)	−0.01 (−0.05, 0.04)	0.738
	Week 12	1.06 (0.25)	1.04 (0.22)		
Vitamin B9, ng/mL	Baseline	9.49 (5.06)	9.49 (5.05)	2.27 (1.37, 3.16)	<0.0001
	Week 12	11.71 (4.79)	13.98 (5.86)		
Vitamin B12, pg/mL ^a^	Baseline	378.95 (284.07, 510.99)	357.84 (238.98, 461.68)	0.02 (−0.01, 0.04)	0.174
	Week 12	369.20 (281.27, 506.65)	371.07 (275.55, 485.98)		
Homocysteine, mmol/L	Baseline	19.87 (7.36)	21.83 (8.42)	−1.24 (−1.94, −0.54)	0.0005
	Week 12	13.57 (3.93)	12.93 (4.02)		
25(OH)D_3_, ng/mL	Baseline	29.81 (8.94)	28.15 (8.38)	−0.23 (−1.08, 0.63)	0.602
	Week 12	30.10 (6.35)	28.84 (6.88)		
DHA, %	Baseline	5.84 (1.15)	5.64 (1.07)	−0.10 (−0.28, 0.08)	0.262
	Week 12	5.88 (1.01)	5.66 (1.07)		
EPA, % ^a^	Baseline	0.42 (0.32, 0.55)	0.37 (0.30, 0.49)	0.09 (0.05, 0.14)	<0.0001
	Week 12	0.49 (0.32, 0.70)	0.52 (0.38, 0.72)		
Calcium, mmol/L	Baseline	1.65 (0.12)	1.67 (0.10)	0.03 (0.004, 0.05)	0.023
	Week 12	1.68 (0.13)	1.71 (0.10)		
Magnesium, mmol/L	Baseline	1.52 (0.15)	1.52 (0.13)	0.01 (−0.02, 0.03)	0.489
	Week 12	1.54 (0.14)	1.54 (0.13)		
Iron, mmol/L	Baseline	8.62 (0.89)	8.70 (0.82)	0.07 (−0.04, 0.18)	0.205
	Week 12	8.66 (0.70)	8.78 (0.71)		
Zinc, μmol/L	Baseline	98.37 (10.97)	100.80 (10.65)	1.59 (−0.39, 3.57)	0.115
	Week 12	99.49 (10.21)	102.24 (10.33)		
Copper, μmol/L	Baseline	16.71 (1.91)	16.53 (1.89)	−0.01 (−0.38, 0.37)	0.978
	Week 12	16.87 (1.53)	16.84 (1.86)		

*Data are presented as mean (standard deviation), or median (1st quartile, 3rd quartile) for non-normal and highly skewed data*.

*Unless otherwise stated, intergroup difference at week 12 was evaluated using analysis of covariance (ANCOVA) adjusted for baseline values*.

a *Intergroup difference at week 12 was evaluated using ANCOVA on log10 of the original data, adjusted for baseline values. Difference of least squares (LS) mean and 95% confidence interval (CI) are presented on a log10 scale*.

*ALT, alanine aminotransferase; AST, aspartate aminotransferase; DHA, docosahexaenoic acid; EPA, eicosapentaenoic acid; HDL, high-density lipoprotein; LDL, low-density lipoprotein; TC, total cholesterol; TG, triglycerides*.

### Effect of PN Intervention on Dietary Intake

**Figure 4:**
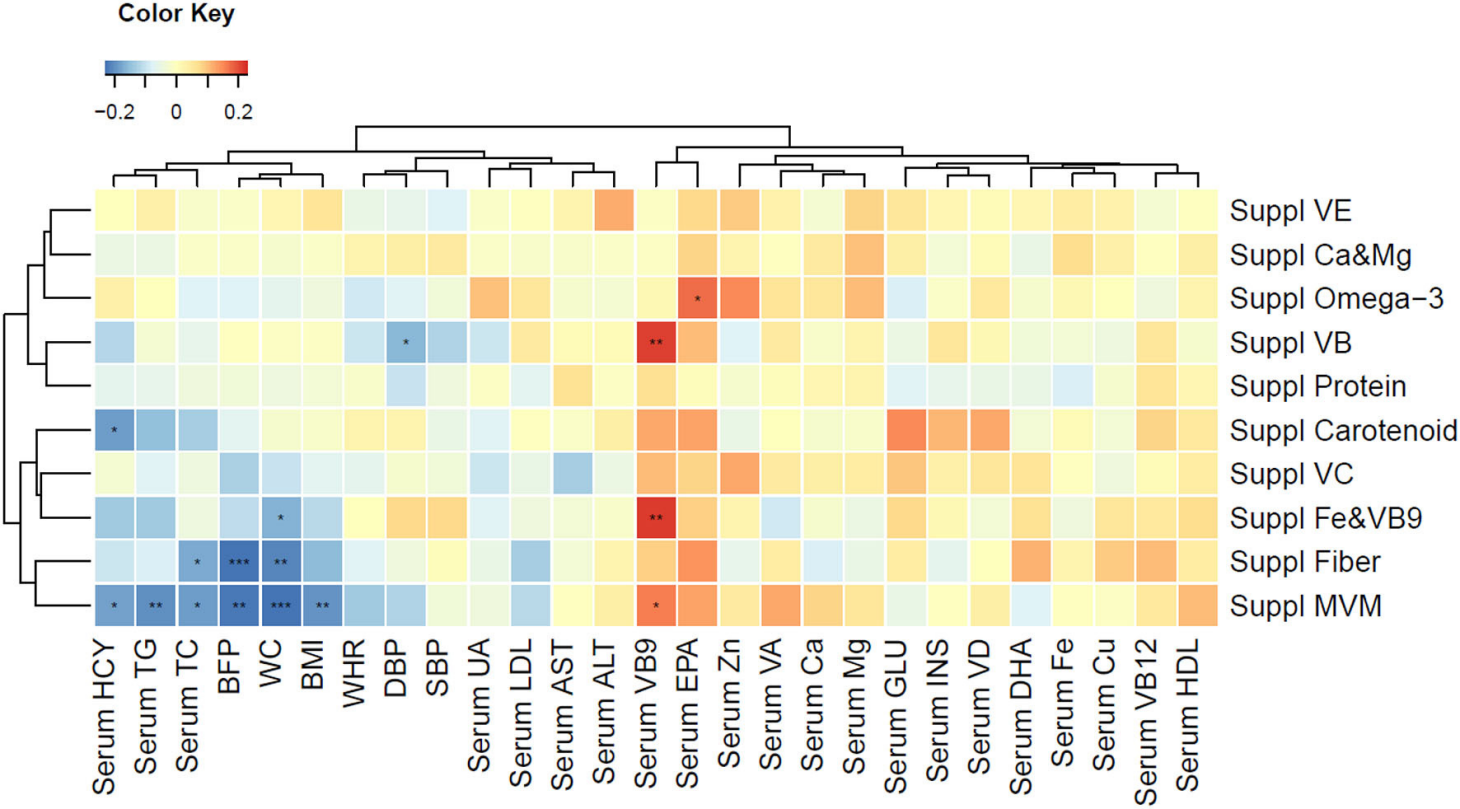
Spearman correlation with clustered clinical outcomes (anthropometric measurements and blood biomarkers) and supplement usage. Blue color indicates strong reverse correlation; red color indicates strong positive correlation. *P < 0.05, **P < 0.01, ***P < 0.001. ALT, alanine aminotransferase; AST, aspartate aminotransferase; BFP, body fat percentage; BMI, body mass index; Ca, calcium; Cu, copper; DBP, diastolic blood pressure; DHA, docosahexaenoic acid; EPA, eicosapentaenoic acid; Fe, iron; GLU, glucose; HCY, homocysteine; HDL, high density lipoprotein; INS, insulin; LDL, low density lipoprotein; Mg, magnesium; MVM, multiple vitamin mineral; SBP, systolic blood pressure; TC, total cholesterol; TG, triglycerides; SBP, systolic blood pressure; Suppl, supplement; UA, uric acid; VA, vitamin A; VB, vitamin B; VC, vitamin C; VD, vitamin D; VE, vitamin E; WC, waist circumference; WHR, waist-to-hip ratio; Zn, zinc.

PN intervention significantly improved the overall diet quality of the subjects, as reflected in the CDGI (Table 3). Compared with the subjects in CG, the subjects receiving PN intervention consumed more whole grains, vegetables, especially dark green vegetables, fruits, dairy products, and nuts. Within-intervention comparison showed the improvement of diet quality in both groups, but PNG showed greater improvement (Supplementary Table 5). Although the energy intake was not signficantly differed, compared with the CG, the PNG had substantially reduced fat consumption and increased dietary intake of beneficial vitamin and minerals (Supplementary Table 6). The dietary modification partially explained why we observed significantly higher circulating EPA, folic acid and calcium levels in the PNG than CG (Table 2). Dietary supplement intake was beneficial in improving blood biomarkers, and Multi-Vitamin and Minerals (MVM) was the only supplement that led to significant BMI decrease (Figure 4).

**Table 3:** Dietary intake and physical activity at baseline and week 12 in both groups.

		**CG** ***N* = 152**	**PNG** ***N* = 166**	**Between-group difference**
				***P*-value**
**Diet**				
Energy, kcal/d	Baseline	1491.5 (1168.5, 1947.0)	1481.0 (1158.0, 1959.0)	0.485
	Week 12	1481.5 (1137.0, 1905.0)	1411.0 (1079.0, 1767.0)	
Whole grain, g/d	Baseline	7.1 (0.0, 28.6)	8.6 (0.0, 28.6)	<0.0001
	Week 12	12.0 (0.0, 46.4)	35.7 (14.3, 57.1)	
Vegetables, g/d	Baseline	187.1 (111.8, 315.0)	191.4 (100.0, 365.7)	<0.0001
	Week 12	262.9 (162.1, 380.7)	323.4 (257.1, 402.9)	
Dark green vegetables, g/d	Baseline	128.6 (71.4, 214.3)	137.9 (64.3, 228.6)	<0.0001
	Week 12	201.8 (109.3, 296.4)	260.0 (194.3, 337.1)	
Fruits, g/d	Baseline	127.1 (57.1, 228.6)	107.1 (57.1, 200.0)	<0.0001
	Week 12	133.9 (56.4, 264.3)	221.4 (146.4, 303.6)	
Dairy products, g/d	Baseline	114.3 (34.3, 250.0)	142.9 (47.1, 235.7)	0.002
	Week 12	128.6 (57.1, 242.9)	200.0 (85.7, 292.9)	
Red meat, g/d	Baseline	57.1 (28.6, 100.0)	64.3 (32.9, 114.3)	0.614
	Week 12	57.1 (29.3, 392.9)	53.2 (32.1, 81.4)	
Nuts, g/d	Baseline	0 (0, 15.7)	0 (0, 214.3)	0.014
	Week 12	0 (0, 13.6)	7.1 (0, 20.0)	
Salt, g/d	Baseline	8.0 (6.0, 10.0)	7.0 (5.0, 12.0)	0.484
	Week 12	6.0 (5.0, 10.0)	6.0 (4.3, 8.0)	
CDGI	Baseline	55.7 (11.7)	55.7 (11.4)	<0.0001
	Week 12	58.6 (12.2)	67.0 (10.9)	
**Physical activity**				
Vigorous MET, min/w	Baseline	0 (0, 480)	0 (0, 960)	0.0009
	Week 12	0 (0, 0)	0 (0, 960)	
Moderate MET, min/w	Baseline	320 (0, 960)	360 (0, 1080)	<0.0001
	Week 12	240 (0, 840)	720 (240, 1680)	
Walking MET, min/w	Baseline	1386 (693, 2079)	1386 (693, 1485)	0.372
	Week 12	1040 (693, 1601)	1386 (693, 1733)	
Total MET, min/w	Baseline	2418 (1386, 3359)	2127 (1215, 3564)	<0.0001
	Week 12	1695 (962, 3066)	2757 (1392, 4558)	
Sitting duration, h/d	Baseline	5 (0, 13)	5 (0, 13)	0.042
	Week 12	5.75 (0, 12.5)	5 (0.08, 12)	
Sleeping duration, h/d	Baseline	7 (3, 12)	7 (3, 10)	0.389
	Week 12	7 (1, 10)	7 (1, 10)	

*Data are presented as median (1st quartile, 3rd quartile) or mean (standard deviation)*.

*Intergroup differences at week 12 were evaluated using Wilcoxon–Mann–Whitney test or ANCOVA adjusted for baseline values*.

*CDGI, China dietary guidelines index; MET, metabolic equivalents of task*.

### Effect of PN Intervention on PA

Although both groups expereinced improvement in PA with the interventions, the PNG had significantly higher vigorous, moderate, and total metabolic equivalent of task (MET), as compared to the CG (Table 3, Supplementary Table 7). In addition, the sitting duration was shortened to a greater extent in the PNG than the CG, whereas the sleeping duration change in both groups was similar. The PA was categorized into three levels (low, moderate, and high); a significantly higher level of PA was observed in PNG after the intervention compared with CG (Supplementary Table 8). Subjects with higher PA level after the intervention experienced greater reduction in body weight, BMI, and waist circumference (Supplementary Figure 1). Based on the wearable device, the PNG had signficantly more step counts and consumed more energy than the CG (Supplementary Table 9).

### Association Between Genotype and PN Intervention Effect

Data collected at the baseline showed significant correlations between the risk of *MTHFR* and blood level of folic acid; the risk of *FTO* and the levels of HDL and uric acid; the risk of *APOE* and the levels of TG, TC and LDL; the risk of *FADS1* and the level of HDL; and the risk of *TCF7L2* and the level of uric acid (Supplementary Table 10). The effects of PN intervention on the change from baseline in anthropometric characteristics, including body weight, BMI, waist circumference, and body fat percent, as well as on the changes in blood levels of TG, TC, HDL, and LDL, appeared to bring more benefits to the subjects carrying risk alleles of *FTO, APOE*, FADS1, and TCF7L2 genes than to those with normal alleles, though none of the interactions between the genotype and intervention group were significant (Supplementary Tables 11, 12).

### PN Intervention Improved Clinical Outcomes by Changing the Behavior

**Figure 5:**
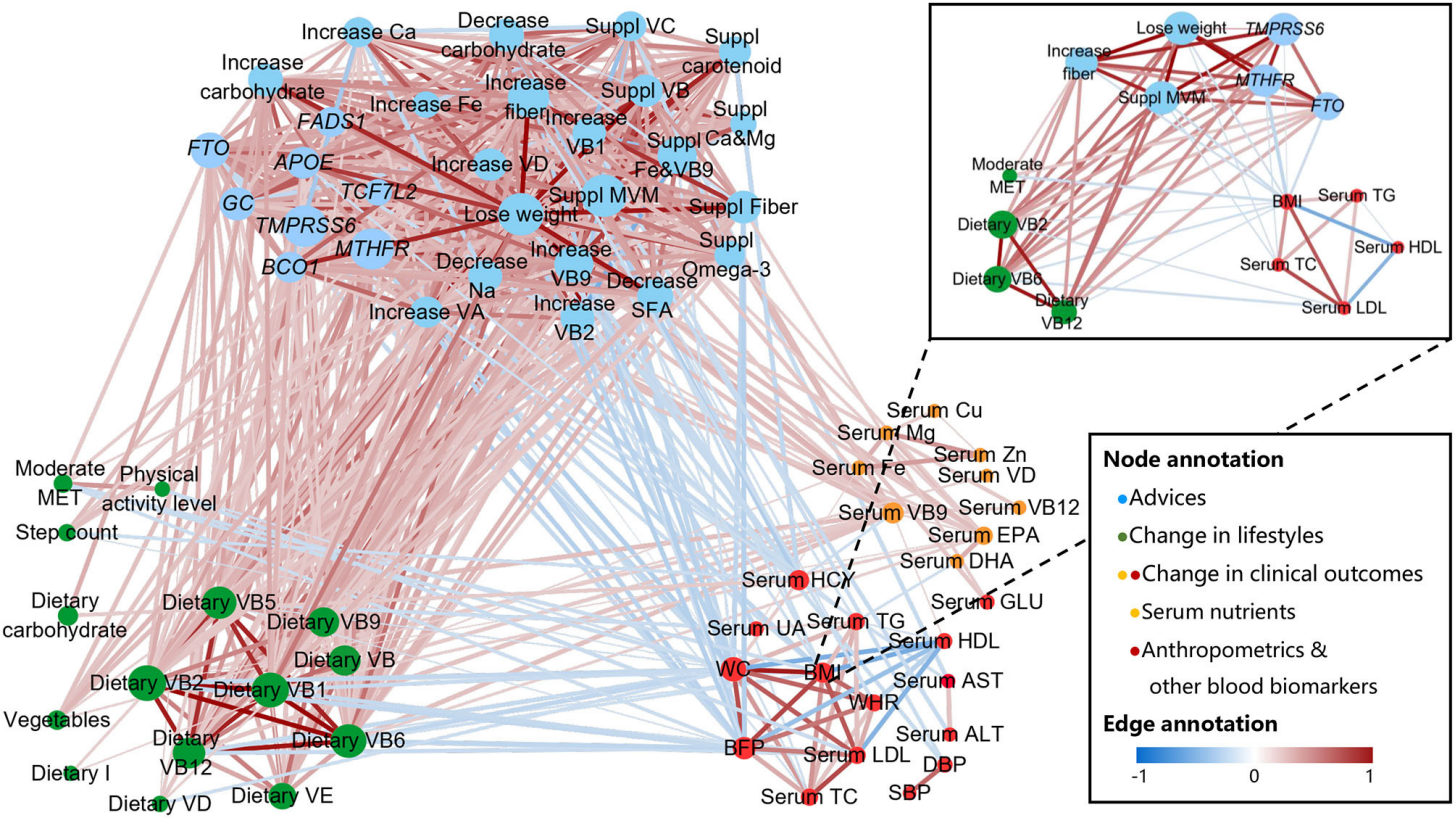
Longitudinal correlation network of advice, genotype, lifestyles, and clinical outcomes. Connections were calculated by Spearman or Point-Biserial with Benjamini-Hochberg-corrected *P* < 0.05 and then plotted as a network by Cytoscape. Nodes are colored according to (1) group of advice, including genetic risk notification (e.g., *FTO*), lifestyle guidance (e.g., lose weight), and nutritional supplements (e.g., Suppl MVM), (2) change in lifestyles (diet and PA), and (3) change in clinical outcomes (anthropometric measurements and blood biomarkers). Node sizes are proportional to betweenness centrality. Edge weights are proportional to the correlation strength, and colors correspond to the direction of association (positive: red; negative: blue). Sub-network in top right shows an example of how “BMI” change be associated with the advice, genotype, lifestyles, and clinical outcomes. ALT, alanine aminotransferase; AST, aspartate aminotransferase; BFP, body fat percentage; BMI, body mass index; Ca, calcium; Cu, copper; DBP, diastolic blood pressure; DHA, docosahexaenoic acid; EPA, eicosapentaenoic acid; Fe, iron; GLU, glucose; HCY, homocysteine; HDL, high density lipoprotein; I, iodine; LDL, low density lipoprotein; MET, metabolic equivalent of task; Mg, magnesium; MVM, multiple vitamin mineral; Na, sodium; SBP, systolic blood pressure; SFA, saturated fatty acid; TC, total cholesterol; TG, triglycerides; UA, uric acid; VA, vitamin A; VB, vitamin B; VC, vitamin C; VD, vitamin D; VE, vitamin E; WC, waist circumference; WHR, waist-to-hip ratio; Zn, zinc.

We analyzed the longitudinal correlation network among the advice in the PN report, changes in lifestyle, and changes in clinical outcomes. Following the given advice, the subjects in PNG increased their PA and optimized nutrient intake, which led to the increase of beneficial nutrients in the blood and to the decrease of “bad” blood biomarkers and anthropometrics (Figure 5). In the PNG, the decrease of BMI was mostly related to the personalized recommendations of “increase fiber,” “lose weight”, and “use MVM supplements,” the increase of moderate MET, and the intake of dietary vitamin B2, B6, and B12. In addition, *TMPRSS6, FTO* and *MTHFR* genotypes mediated the efficacy of nutritional recommendations and lifestyle modifications on BMI decrease (Figure 5, sub-network).

## Discussion

Health benefits achieved by PN should be measured using validated clinical outcomes, such as anthropometric parameters (e.g., BMI, body fat percentage, and waist circumference) and blood biomarkers (e.g., blood lipids, blood glucose, and blood nutrients) (10). Anthropometric measurements and blood biomarkers are suitable for PN assessment and monitoring as they provide real-time information that reflects an individual's current metabolic or physiological state (6). More importantly, the information is readily actionable and easily trackable, as these parameters change over time in response to diet, PA, and other lifestyle factors (6). In this study, we observed improved clinical outcomes (weight, BMI, body fat percentage, waist circumference, waist-to-hip ratio, blood TG, TC, LDL, uric acid, homocysteine, folic acid, EPA, and calcium) in the PNG compared with those in the CG. This was the first RCT to show the health benefits of PN intervention in overweight/obese Chinese adults. In the Food4Me study, there was no significant difference in clinical outcomes between PN and control groups, though a trend was observed in weight, BMI, waist circumference, and blood TC (5). The reason might be the lack of inclusion criteria on BMI in the Food4Me study, while in this study we included overweight/obese adults because there is evidence that people are more open to health-promoting information when the individuals recognize themselves as being highly susceptible to preventable diseases (9, 30).

Diet is among the most important modifiable lifestyle factors contributing to NCDs risk (9). Diets with high intake of energy-dense and highly refined carbohydrate foods are associated with obesity and type 2 diabetes (31). Changes in diet, especially a personalized diet, can prevent the onset of disease (9). Zeevi et al. continuously monitored glucose levels in an 800-person cohort, measured responses to 46,898 meals, and found high variability in the response to identical meals, suggesting that universal dietary recommendations may have limited utility (7). In the PREDICT1 study, a machine-learning model was created to predict both triglyceride and glycemic responses to food intake for developing personalized diet strategies (4). These leading-edge studies shed new light on the personalized diet intervention. In a previous RCT on PN, changing dietary intake to better align with the MedDiet—widely recognized as a healthy eating pattern—produced substantial health benefits (8). The participants who received PN intervention consumed less red meat, salt, and saturated fat, and had higher healthy eating index score (5). In our study, we observed similar dietary behavior changes in PNG, including increased consumption of whole grains, vegetables, especially dark green vegetables, fruits, dairy products, and nuts, and an improved CDGI, which again, validated that PN intervention is more efficacious than the conventional nutrition intervention. One of the novelties of the current study is the application of dietary supplements, especially among the subjects with high risk of deficiencies. Among all the supplements, MVM displayed the most potent efficacy by modulating serum TG, TC, homocysteine, and folic acid, and facilitating the decrease in BMI, body fat percentage and waist circumference. This was within expectation since MVM offered a wide range of nutrients, which might generate synergistic effects in promoting health outcomes. MVM would be an optimal choice for the overweight/obese adults who aim to lose weight.

In addition to diet, PA is another important modifiable lifestyle factor included in PN intervention (32). Sedentary behavior and physical inactivity are among the leading modifiable risk factors worldwide for cardiovascular disease and all-cause mortality (33). For all-cause mortality, spending > 3–4 h/day in watching television and > 6–8 h/day in any sitting activity have been suggested as detrimental (34). Although the health benefits of PA and exercise are evident, the research on personalized PA intervention is still limited. The Food4Me study showed that PA attenuated the effect of the *FTO* genotype on obesity traits in European adults (12). It is important to note that, subjects in the CG and PNG recieved the exercise notifications at the same frequency, so the differences in MET were not due to the subjects' awareness. Previous RCTs have shown that tailored training, or personal training, are superior to untailored trainging in promoting PA (35, 36), potentially through changing the participants' attitudes in PA. In this case, the subjects in the PNG might feel more motivated when they read the tailored notifications, which led to promoted PA duration and intensity. These findings suggested the important contribution of individualized PA advice to PN intervention.

PN advice promotes changes in individual dietary and PA behaviors, which may result in health or function improvement (10). In the P100 study, specific recommendations based on personal data were customized by a coach to help participants modify their behaviors, which potentially improved their clinical biomarkers (37). The Food4Me study successfully led to health-related behavioral changes but failed to bring significant improvement in clinical outcomes (5). In our study, following PN advice, the subjects improved their PA and nutrient intake, which was associated with the increase of beneficial nutrients in the blood and the decrease of “bad” blood biomarkers and anthropometrics. For instance, BMI decrease was associated with the increase of moderate MET, and the intake of dietary vitamin B2, B6, and B12. The results suggested that our PN intervention improved the clinical outcomes by changing the subjects' behavior. An important element to consider relating to the efficacy of PN intervention is the sustainability of behavior change, or longer-term adherence to personalized diet and lifestyle recommendations (10). That would require a precise and validated algorithm, professional and trustworthy guidance from registered dietitian, and compassionate and sustainable communication with a service team.

According to several publications, the PN intervention strategies that include genetic information have greater potential than the ones based on the phenotype alone for improving health (38–40). Adding a genetic component and disclosure of genetic information may bring crisis awareness and improve motivation and compliance (38). In addition, people who carry risk alleles may develop certain nutrient deficiency even when their intake meets recommended levels (41, 42). Since the efficacy of diet could be modulated or impaired by the SNPs of certain enzymes (43), it is critical to apply dietary supplements among the population that are genetically at risk of deficiencies. Although the PN intervention effects on the changes in anthropometric characteristics and blood biomarkers were similar for subjects with risk or non-risk allele of *FTO, APOE, FADS1*, and *TCF7L2* genes, we still observed that subjects with a risk allele experienced greater improvement in certain clinical outcomes. Follow up studies are warranted in the future to further explore the association between genotype and PN intervention effect.

As expected, the decrease of BMI was significantly associated with the “lose weight” advice, since a series of suggestions were provided under the “lose weight” node, which included dietary changes and PA modifications, the most critical modifiable lifestyle factors that are related to weight loss (44). The B complex vitamins play an important role in maintaining energy homeostasis. Vitamin B2, for example, is a vital cofactor within the electron transport chain, Krebs cycle, and beta-oxidation (fat burning) (45), which explained why the increased vitamin B2 intake is a major contributor of BMI reduction. Vitamin B6 and vitamin B12 are involved in one-carbon metabolism, in which MTHFR plays a critical role (46). Although there is a lack of clear evidence of how promoted one-carbon metabolism is related to weight loss, it is reported that vitamin B intake is positively associated with fat-free mass in overweight/obese females (47), and according to a meta-analysis with 9,075 participants, higher serum vitamin B12 levels are inversely associated with obesity (48). Expectedly, as the first identified obesity-related gene, *FTO* polymorphisms affected weight loss. As an RNA N^6^-methyladenosine (m^6^A) demethylase, the group of FTO proteins are described as a regulator of m^6^A level of hormones, such as ghrelin, to modify energy intake and adipogenesis (23, 49). In addition to *FTO* and *MTHFR, TMPRSS6* polymorphism is another key genetic factor in the longitudinal correlation network. This is interesting since no evidence has shown that *TMPRSS6* affects energy metabolism. As reported, *TMPRSS6* genotype influences iron metabolism, and the mutations in *TMPRSS6* may lead to iron deficiency (27, 50). Previous studies have shown that a low iron level may result in exercise intolerance, weakness, and impaired muscle strength (51, 52). Iron supplementation in the iron-depleted females significantly improved their progressive fatigue resistance during exercise (53). Considering that the increase in moderate MET is one of the major contributors to BMI decrease, it is possible that the subjects with *TMPRSS6* risk alleles may have experienced more fatigue during exercises, which potentially affected their PA duration and intensity, and subsequently sabotaged their plan on weight loss.

Admittedly, there are several limitations in this study. PN intervention is a holistic and integrated solution, in which continuous follow-up action and motivation are part of the whole PN intervention strategy to help the subjects achieve the goal (54). In this study, we cautiously designed the intervention of CG and PNG to minimize bias between the groups. Nevertheless, we admit that the intensity of intervention cannot be completely consistent between the groups, which is due to the nature of PN intervention (15, 54). Another limitation is the lack of objective biomarkers for assessing adherence. Unlike non-personalized dietary intervention trials, it is difficult to identify certain objective biomarker(s) to fully indicate the adherence in the personalized setting. As alternatives to objective biomarkers, CDGI, step count, and pill count were used in our study to show the compliance of diet, PA, and nutritional supplements, respectively. Previous studies have shown that microbiota phenotypes might modulate physiological responses to diet, especially postprandial TG, glucose, and insulin (4, 7). The analysis of “omics” data, such as metagenomics and metabolomics data, may provide a broad perspective on the changes of large sets of biomarkers (4, 55). In the near future, we will add more parameters to the PN report by including the multi-omics data.

Our current study has advanced our knowledge from previously published PN studies. It is important to note that this is the first RCT in China that showed the health benefits of PN intervention in overweight/obese adults, which provided a model of framework for developing the personalized advice that leads to modification of lifestyle and improvement of clinical outcomes. In previous studies, nutritional supplements were usually neglected by investigators, even though taking supplements is suggested when the residents are limiting dietary intake (56), which leaves a major gap of knowledge in the study of PN intervention. In contrast, we provided a holistic and integrated solution, containing both diet (supplements were used as a tool to fill the gap) and PA, to each individual in the PNG. Moreover, we combined two critical elements in the delivery of PN report: the validated decision trees as an algorithm to develop a PN report and the registered dietitians certificated to provide the solution. Currently, we are coding the decision trees to develop a mini application to carry out a real-world study, which may expand our data sources and facilitate our understanding on the efficacy of PN intervention in everyday environments.

In conclusion, we demonstrated that PN intervention showed greater benefits to health status in overweight/obese Chinese adults compared with the benefits from conventional intervention. PN intervention improved the clinical outcomes of anthropometric characteristics and blood biomarkers by changing the dietary and PA behaviors of subjects. The approach and the associated outcomes presented here open up the possibility for positive health outcomes in a general population should a similar program become widely available.

## Data Availability Statement

The original contributions presented in the study are included in the article/Supplementary Material, further inquiries can be directed to the corresponding author/s.

## Ethics Statement

The studies involving human participants were reviewed and approved by the Institutional Review Board (IRB) of the Shanghai Nutrition Society. The participants provided their written informed consent to participate in this study.

## Author Contributions

JK, HG, and JD designed the research. JK, KX, and PW conducted the research. JN, FW, JC, and JZ analyzed the data. JK and JC wrote the manuscript. JK, JC, MR, JN, FW, and JZ revised the manuscript. JD and HG had primary responsibility for final content. All authors read and approved the final manuscript.

## Conflict of Interest

The authors declare that this study received funding from Amway. The funder was not involved in the study design, collection, analysis, interpretation of data, the writing of this article or the decision to submit it for publication.

## Publisher's Note

All claims expressed in this article are solely those of the authors and do not necessarily represent those of their affiliated organizations, or those of the publisher, the editors and the reviewers. Any product that may be evaluated in this article, or claim that may be made by its manufacturer, is not guaranteed or endorsed by the publisher.
