# Supplementary material for: Personalized Nutrition Intervention Improves Health Status in Overweight/Obese Chinese Adults: A Randomized Controlled Trial
Source: Front Nutr. 2022 Jun 22;9:919882. doi: 10.3389/fnut.2022.919882 (PMC9258630; doi:10.3389/fnut.2022.919882)
Supplement: Supplementary file 1 [file Data_Sheet_1.docx]

**Supplementary Table 1. Product list**

| **Product Name** | **Supplemented Nutrient** | **Dosage Form** | **SKU Number** |
| --- | --- | --- | --- |
| Salmon Omega-3 | Omega-3 | Soft gel | 20508 |
| All Plant Protein Powder | Protein | Powder | 20495 |
| Double X | Multi-vitamin and minerals | Tablet | 20499 |
| Fruit & Vegetable Fiber | Fiber | Chewable tablet | 20509 |
| Calcium Magnesium | Calcium/magnesium | Tablet | 20504 |
| Iron-Folic Plus | Iron/folic acid | Tablet | 20513 |
| Natural B Complex | Vitamin B | Tablet | 20502 |
| Bio C Plus | Vitamin C | Tablet | 20505 |
| Natural Multi Carotene | Carotenoid | Soft gel | 20511 |
| Wheat Gem E | Vitamin E | Soft gel | 20507 |

**Supplementary Table 2. Gene list**

| **Target** | **Gene** | **Protein** | **RS Number** | **Common Allele** | **Risk Allele** | **Risk Genotype** |
| --- | --- | --- | --- | --- | --- | --- |
| Vitamin A | *BCO1* | β-carotene-15, 15'-momooxygenase 1 | rs12934922 | A | T | AT, TT |
| Vitamin D | *GC* | Vitamin D-binding protein | rs4588 | C | A | AA |
| Vitamin B | *MTHFR* | 5,10-methylenetetrahydrofolate reductase | rs1801133 | C | T | TT, CT |
| Lipid | *FTO* |  | rs1121980 | C | T | TC, TT |
|  | *APOE* | Apolipoprotein E | rs769449 | G | A | AA / CC, AA / CT, AG/ CC, AG/ CT |
|  |  |  | rs7412 | T | C |  |
| DHA&EPA | *FADS1* | Fatty acid desaturase | rs174546 | C | T | TT |
| Glucose | *TCF7L2* | Transcription factor 7-like 2 | rs7903146 | C | T | TT, CT |
| Calcium | *CaSR* | Calcium-sensing receptor | rs17251221 | A | G | GG, AG |
| Iron | *TMPRSS6* | Transmembrane serine protease 6 | rs855791 | C | T | CT, TT |

**Supplementary Table 3. Within-intervention comparison of anthropometrics**

|  | **CG**  **N = 152** | |  | **PNG**  **N = 166** | |
| --- | --- | --- | --- | --- | --- |
|  | **Mean Difference (95% CI)** | **p-value** |  | **Mean Difference (95% CI)** | **p-value** |
| Weight (kg) | -1.21 (-1.61, -0.82) | <0.0001 |  | -2.13 (-2.51, -1.75) | <0.0001 |
| Height (cm) | 0.06 (-0.02, 0.13) | 0.140 |  | 0.01 (-0.07, 0.10) | 0.756 |
| BMI (kg/m^2^) | -0.48 (-0.63, -0.32) | <0.0001 |  | -0.80 (-0.94, -0.66) | <0.0001 |
| Body fat percentage (%) | -0.48 (-0.68, -0.29) | <0.0001 |  | -1.23 (-1.47, -1.00) | <0.0001 |
| Waist circumference (cm) | -1.68 (-2.04, -1.32) | <0.0001 |  | -2.87 (-3.22, -2.51) | <0.0001 |
| Waist-to-hip ratio | -0.01 (-0.02, 0.01) | <0.0001 |  | -0.021 (-0.027, -0.015) | <0.0001 |
| Systolic pressure (mmHg) | 0.78 (-1.14, 2.71) | 0.423 |  | 1.89 (-0.32, 4.10) | 0.093 |
| Diastolic pressure (mmHg) | 1.07 (-0.29, 2.42) | 0.123 |  | 0.26 (-1.63, 2.15) | 0.787 |

Data presented are mean difference (95% confidence interval) between week 12 and baseline measurements in each intervention arm. Within-intervention comparison was performed using paired t-test. CI: confidence interval

**Supplementary Table 4. Within-intervention comparison of blood biomarkers**

|  | **CG**  **N = 152** | |  | **PNG**  **N = 166** | |
| --- | --- | --- | --- | --- | --- |
|  | **Within Group Change** | ***P* value** |  | **Within Group Change** | ***P* value** |
| Triglyceride (TG), mmol/L | -0.09 (0.38) | <0.0001 ^a^ |  | -0.23 (0.35) | <0.0001 ^a^ |
| Total cholesterol (TC), mmol/L | -0.14 (-0.23, -0.05) | 0.002 |  | -0.31 (-0.37, -0.24) | <0.0001 |
| High-density lipoprotein (HDL), mmol/L | 0.08 (0.04, 0.11) | <0.0001 |  | 0.12 (0.09, 0.14) | <0.0001 |
| Low-density lipoprotein (LDL), mmol/L | -0.26 (-0.33, -0.19) | <0.0001 |  | -0.36 (-0.42, -0.31) | <0.0001 |
| Glucose, mmol/L | -0.20 (0.70) | 0.001 ^b^ |  | -0.20 (0.60) | <0.0001 ^b^ |
| Alanine aminotransferase (ALT), U/L | 1.20 (29.70) | 0.541 ^a^ |  | -0.25 (14.60) | 0.863 ^a^ |
| Aspartate aminotransferase (AST), U/L | 1.05 (19.85) | 0.739 ^a^ |  | -0.95 (7.90) | 0.638 ^a^ |
| Uric acid, mmol/L | -7.42 (-14.47, -0.37) | 0.039 |  | -20.23 (-27.88, -12.57) | <0.0001 |
| Docosahexaenoic acid (DHA), % | 0.04 (-0.11, 0.19) | 0.586 |  | 0.02 (-0.12, 0.16) | 0.866 |
| Eicosapentaenoic acid (EPA), % | 0.04 (0.32) | 0.188 ^a^ |  | 0.12 (0.25) | <0.0001 |
| Insulin, μU/ml | -0.41 (7.20) | 0.433 ^a^ |  | -0.51 (5.90) | 0.053 ^a^ |
| Vitamin A, mg/L | 0.02 (-0.03, 0.07) | 0.390 |  | 0.04 (-0.004, 0.08) | 0.078 |
| Vitamin B9 (Folate), ng/ml | 2.22 (1.57, 2.87) | <0.0001 |  | 4.49 (3.79, 5.18) | <0.0001 |
| Vitamin B12, pg/ml | 11.59 (141.29) | 0.297 ^a^ |  | 21.96 (98.82) | 0.117 ^a^ |
| Vitamin 25(OH)D3, ng/ml | 0.29 (-0.56, 1.14) | 0.500 |  | 0.69 (-0.05, 1.42) | 0.066 |
| Homocysteine (HCY), mmol/L | -6.30 (-7.28, -5.31) | <0.0001 |  | -8.90 (-9.89, -7.90) | <0.0001 |
| Calcium (Ca), mmol/L | 0.02 (-0.003, 0.05) | 0.077 |  | 0.04 (0.02, 0.06) | 0.0005 |
| Magnesium (Mg), mmol/L | 0.02 (-0.003, 0.04) | 0.090 |  | 0.03 (0.01, 0.05) | 0.004 |
| Iron (Fe), mmol/L | 0.04 (-0.06, 0.148) | 0.433 |  | 0.08 (-0.004, 0.16) | 0.061 |
| Zinc (Zn), μmol /L | 1.12 (-0.69, 2.92) | 0.225 |  | 1.44 (-0.06, 2.93) | 0.060 |
| Copper (Cu), μmol/L | 0.16 (-0.25, 0.56) | 0.441 |  | 0.32 (-0.03, 0.66) | 0.072 |

Data presented are mean difference (95% confidence interval) or median difference (interquartile range) for non-normal and highly skewed data between week 12 and baseline. Interquartile range is the difference between the 3^rd^ and the 1^st^quartile.

Unless otherwise stated, within-intervention comparison was performed using paired t-test.

^a.^ Within-intervention comparison was performed using paired t-test on log 10 of the original data.

^b.^ Within-intervention comparison was performed using Wilcoxon signed ranks test.

**Supplementary Table 5. Within-intervention comparison of dietary intake**

|  | **CG**  **N = 152** | |  | **PNG**  **N = 166** | |
| --- | --- | --- | --- | --- | --- |
|  | **Within Group Change** | **Signed Rank**  ***P* value** |  | **Within Group Change** | **Signed Rank**  ***P* value** |
| Cereals, g/day | -13.2 (158.1) | 0.063 |  | 17.9 (201.0) | 0.634 |
| Whole grain | 0.0 (34.3) | 0.006 |  | 13.6 (44.3) | <0.0001 |
| Soybeans, g/day | 0.0 (93.2) | 0.712 |  | 14.3 (108.6) | 0.186 |
| Vegetables, g/day | 19.6 (227.1) | 0.022 |  | 93.2 (166.4) | <0.0001 |
| Dark vegetables | 32.1 (163.9) | <0.0001 |  | 104.3 (144.3) | <0.0001 |
| Mushrooms and seaweeds, g/day | -5.0 (30.5) | 0.011 |  | -1.8 (36.4) | 0.069 |
| Fruits, g/day | -15.0 (144.6) | 0.828 |  | 64.3 (142.9) | <0.0001 |
| Dairy product, g/day | -5.0 (130.7) | 0.900 |  | 20.7 (151.4) | 0.002 |
| Meat, g/day | -4.3 (104.3) | 0.187 |  | -22.1 (109.3) | <0.0001 |
| Red meat | 0.0 (74.7) | 0.457 |  | -14.3 (65.3) | <0.0001 |
| Aquatic products, g/day | -7.1 (85.7) | 0.377 |  | 2.5 (86.4) | 0.374 |
| Eggs, g/day | -3.2 (38.6) | 0.200 |  | 4.3 (42.9) | 0.108 |
| Snacks, g/day | -2.5 (53.2) | 0.058 |  | 0.0 (64.3) | 0.464 |
| Nuts | 0.0 (10.0) | 0.205 |  | 0.0 (13.6) | 0.008 |
| Beverages, g/day | -0.7 (296.4) | 0.096 |  | 0.0 (245.7) | 0.264 |
| Condiment, g/day | -12.7 (48.8) | 0.0003 |  | -10.9 (45.7) | <0.0001 |
| Oil | -5.0 (30.0) | 0.017 |  | -4.0 (22.1) | 0.002 |
| Salt | -0.9 (9.6) | 0.127 |  | -1.9 (6.1) | <0.0001 |
| Cigarettes, number/day | 0.0 (0.0) | 0.534 |  | 0.0 (0.0) | 0.978 |
| Alcohols, g/day | 0.0 (0.0) | 0.109 |  | 0.0 (0.0) | 0.079 |
| China dietary guidelines index | 2.9 (0.7, 5.1) | 0.011 |  | 11.3 (9.4, 13.2) | <0.0001 |

Data shown are mean difference (95% confidence interval) and median difference (interquartile range) between week 12 and baseline. Interquartile range is the difference between the 3^rd^ and the 1^st^ quartile. Within-group difference from baseline to week 12 are evaluated using Wilcoxon signed ranks test.

**Supplementary Table 6. Summary of daily energy and nutrients intake**

|  | **Baseline ^a^** | | |  | **Week 12 ^a^** | | |  | **Week 12 vs. Baseline ^b^** | | | |
| --- | --- | --- | --- | --- | --- | --- | --- | --- | --- | --- | --- | --- |
|  | **CG**  **N = 152** | **PNG**  **N = 166** | **Group Difference *P* value** |  | **CG**  **N = 152** | **PNG**  **N = 166** | **Group Difference *P* value** |  | **CG** | | **PNG** | |
|  |  |  |  |  |  |  |  |  | **Within Group Change** | ***P* value** | **Within Group Change** | ***P* value** |
| Energy, Kcal | 1491.5 (1168.5-1947.0) | 1481.0 (1158.0-1959.0) | 0.807 |  | 1481.5 (1137.0-1905.0) | 1411.0 (1079.0-1767.0) | 0.485 |  | -18.0 (832.0) | 0.369 | -137.5 (911.0) | 0.157 |
| Protein, g | 62.4 (44.5-86.5) | 64.5 (44.8-87.8) | 0.771 |  | 63.1 (49.8-88.4) | 62.7 (47.2-80.6) | 0.742 |  | -0.1 (41.7) | 0.822 | 0.3 (41.3) | 0.689 |
| Fat, g | 61.8 (43.4-85.0) | 60.1 (40.6-85.8) | 0.378 |  | 56.3 (40.5-78.2) | 49.3 (36.6-69.2) | 0.144 |  | -4.9 (45.4) | 0.032 | -9.2 (50.7) | 0.006 |
| Carbohydrate, g | 162.9 (115.6-222.9) | 161.0 (110.4-230.2) | 0.960 |  | 161.3 (116.6-231.8) | 154.7 (114.0-236.2) | 0.990 |  | -1.6 (127.4) | 0.969 | 1.9 (144.7) | 0.920 |
| Dietary fiber, g | 7.3 (5.1-11.0) | 7.2 (4.5-11.5) | 0.896 |  | 7.2 (4.4-12.6) | 7.5 (4.4-11.3) | 0.429 |  | -0.5 (8.4) | 0.909 | 0.3 (8.3) | 0.274 |
| Vitamin A, μgRE | 311.0 (167.0-484.5) | 298.5 (176.0-516.0) | 0.399 |  | 338.5 (225.5-545.0) | 454.5 (292.0-658.0) | 0.004 |  | 46.0 (404.0) | 0.122 | 136.0 (485.0) | 0.008 |
| Vitamin D, μg ^c^ | 1.1 (0.0-3.0) | 1.2 (0.4-3.4) | 0.225 |  | 1.2 (0.2-3.8) | 5.0 (2.5-6.8) | <0.0001 |  | 0.0 (3.3) | 0.840 | 3.5 (5.5) | <0.0001 |
| Vitamin E, mg | 24.1 (14.0-32.2) | 20.5 (12.4-29.5) | 0.052 |  | 19.1 (11.8-29.5) | 47.8 (29.1-57.4) | <0.0001 |  | -3.1 (26.3) | 0.016 | 23.5 (32.8) | <0.0001 |
| Vitamin B1, mg | 0.7 (0.4-1.0) | 0.6 (0.4-0.9) | 0.701 |  | 0.7 (0.5-1.1) | 5.9 (1.2-6.4) | <0.0001 |  | 0.06 (0.74) | 0.631 | 5.0 (5.5) | <0.0001 |
| Vitamin B2, μg | 0.8 (0.6-1.0) | 0.8 (0.6-1.2) | 0.418 |  | 0.9 (0.6-1.3) | 6.0 (1.3-6.5) | <0.0001 |  | 0.08 (0.60) | 0.049 | 5.2 (5.7) | <0.0001 |
| Vitamin B6, mg ^c^ | 0.1 (0.1-0.3) | 0.1 (0.1-0.2) | 0.590 |  | 0.2 (0.1-0.3) | 5.6 (0.3-5.7) | <0.0001 |  | 0.04 (0.26) | 0.020 | 5.3 (5.4) | <0.0001 |
| Vitamin B12, μg ^c^ | 0.0 (0.0-0.0) | 0.0 (0.0-0.0) | 0.490 |  | 0.0 (0.0-0.0) | 3.8 (0.0-3.8) | <0.0001 |  | 0.0 (0.0) | 0.354 | 3.8 (3.8) | <0.0001 |
| Vitamin C, mg | 59.9 (23.1-93.0) | 53.3 (31.2-91.8) | 0.762 |  | 76.0 (39.1-151.0) | 261.3 (94.6-331.8) | <0.0001 |  | 18.3 (107.5) | 0.505 | 203.0 (262.7) | <0.0001 |
| Vitamin B5 (Pantothenic acid), mg ^c^ | 0.0 (0.0-0.0) | 0.0 (0.0-0.0) | NA |  | 0.0 (0.0-0.0) | 10.0 (0.0-10.0) | <0.0001 |  | 0.0 (0.0) | 0.016 | 10.0 (10.0) | <0.0001 |
| Vitamin B9 (Folate), μg | 77.6 (37.3-134.9) | 71.8 (41.3-138.5) | 0.111 |  | 116.6 (40.9-180.1) | 282.6 (197.4-404.1) | <0.0001 |  | 28.0 (145.7) | 0.053 | 183.8 (191.0) | <0.0001 |
| Vitamin B3 (Niacin), mg | 13.4 (9.1-19.6) | 13.3 (8.9-19.4) | 0.907 |  | 15.2 (10.2-21.3) | 22.9 (15.7-30.2) | <0.0001 |  | 1.8 (12.1) | 0.485 | 6.6 (17.0) | <0.0001 |
| Ca, mg | 410.5 (267.5-600.5) | 412.0 (272.0-647.0) | 0.432 |  | 515.5 (332.5-695.0) | 671.5 (474.0-896.0) | <0.0001 |  | 34.0 (494.0) | 0.189 | 239.0 (506.0) | <0.0001 |
| P, mg | 840.8 (652.1-1178.8) | 848.5 (647.5-1105.3) | 0.903 |  | 908.4 (646.7-1154.1) | 873.6 (642.2-1082.5) | 0.645 |  | 13.0 (599.4) | 0.907 | -2.4 (539.8) | 0.710 |
| Ka, mg | 1586.8 (1113.0-2189.4) | 1474.8 (1093.2-2048.3) | 0.781 |  | 1611.9 (1247.3-2173.6) | 1457.3 (1059.1-1930.7) | 0.237 |  | 15.3 (1233.0) | 0.564 | -43.9 (1120.0) | 0.059 |
| Na, mg | 3720.8 (2264.6-5521.7) | 3809.2 (2461.1-5844.1) | 0.734 |  | 3520.9 (2141.6-5133.8) | 2978.4 (1827.1-4436.2) | 0.077 |  | -173.6 (4176.0) | 0.515 | -668.7 (4012.0) | 0.022 |
| Mg, mg | 245.5 (185.0-337.5) | 221.0 (179.0-288.0) | 0.333 |  | 261.5 (199.5-337.0) | 332.0 (233.0-404.0) | 0.0003 |  | 8.5 (199.5) | 0.768 | 87.0 (221.0) | <0.0001 |
| Fe, mg | 16.2 (12.4-21.9) | 14.4 (11.0-20.2) | 0.322 |  | 16.6 (11.9-22.9) | 18.0 (12.7-24.8) | 0.193 |  | 0.2 (14.5) | 0.909 | 2.5 (11.9) | 0.017 |
| I, μg | 32.1 (18.7-57.9) | 29.5 (17.8-51.6) | 0.949 |  | 30.0 (14.5-51.9) | 30.1 (15.4-52.6) | 0.379 |  | -1.1 (43.9) | 0.035 | -0.6 (47.8) | 0.191 |
| Zn, mg | 8.1 (5.9-10.8) | 7.7 (5.9-10.2) | 0.828 |  | 8.9 (6.5-12.4) | 12.3 (7.7-15.9) | <0.0001 |  | 0.7 (7.0) | 0.370 | 3.1 (10.0) | <0.0001 |
| Se, μg | 41.4 (26.3-72.8) | 40.6 (27.8-67.0) | 0.851 |  | 43.7 (30.7-66.1) | 43.0 (31.9-63.6) | 0.603 |  | 4.4 (47.7) | 0.865 | 3.0 (41.1) | 0.582 |
| Cu, mg | 1.2 (0.8-2.0) | 1.1 (0.8-1.7) | 0.571 |  | 1.1 (0.8-1.9) | 1.5 (1.0-2.2) | 0.007 |  | 0.02 (1.2) | 0.137 | 0.2 (1.4) | 0.079 |
| Mn, mg | 3.0 (2.2-4.9) | 2.6 (1.9-4.2) | 0.297 |  | 3.4 (2.2-5.3) | 4.1 (2.8-5.7) | 0.007 |  | -0.01 (3.7) | 0.995 | 1.2 (3.2) | 0.0002 |
| Choline, mg ^c^ | 18.7 (2.5-41.8) | 22.5 (6.5-46.9) | 0.150 |  | 12.6 (5.3-36.5) | 17.4 (4.4-42.1) | 0.392 |  | 0.0 (35.1) | 0.546 | -2.6 (51.8) | 0.402 |
| Biotin, μg ^c^ | 4.7 (1.6-10.9) | 4.6 (1.7-10.0) | 0.611 |  | 3.9 (1.0-10.4) | 4.8 (2.1-8.5) | 0.143 |  | -0.3 (8.9) | 0.241 | 0.0 (9.1) | 0.737 |

^a^ Data presented are median (1^st^ quartile-3^rd^ quartile). Unless otherwise stated, group differences were evaluated using analysis of variance on log10 of the original data; week 12 model adjusted for baseline log values (except for pantothenic acid, which was not detected at baseline).

^b^ Data shown are median (interquartile range) of the difference between week 12 and baseline. Interquartile range is the difference between the 3^rd^ and the 1^st^ quartile. Within-group changes compared to baseline are evaluated using paired t-test on log transformed data.

^c^ Group differences are evaluated using Wilcoxon-Mann Whitney test for highly skewed data; within group changes are evaluated using Wilcoxon signed ranks test.

**Supplementary Table 7. Within-intervention comparison of physical activity**

|  | **CG**  **N = 152** | |  | **PNG**  **N = 166** | |
| --- | --- | --- | --- | --- | --- |
|  | **Median Difference (Interquartile Range)** | **Signed Rank**  ***P* value** |  | **Median Difference (Interquartile Range)** | **Signed Rank**  ***P* value** |
| Walk MET-minutes/week | 0.00 (1262.0) | 0.111 |  | 0.0 (1188) | 0.789 |
| Moderate MET-minutes/week | 0.00 (700.0) | 0.348 |  | 170.0 (720.0) | <0.0001 |
| Vigorous MET-minutes/week | 0.00 (200.0) | 0.099 |  | 0.0 (80.0) | 0.611 |
| Total MET-minutes/week | -310.5 (2275.0) | 0.040 |  | 253.5 (2106.0) | 0.018 |

Data presented are median difference (interquartile range) between week 12 and baseline. Within-intervention comparison was performed using Wilcoxon signed ranks test. Interquartile range is the difference between the 3^rd^ and the 1^st^ quartile.

**Supplementary Table 8. Differences in physical activity level at baseline and week 12**

|  | **Baseline** | | |  | **Week 12** | | |
| --- | --- | --- | --- | --- | --- | --- | --- |
|  | **CG**  **N = 152** | **PNG**  **N = 166** | ***P* value** |  | **CG**  **N = 152** | **PNG**  **N = 166** | ***P* value** |
| **Level of Physical Activity** |  |  | 0.343 |  |  |  | 0.0003 |
| Low | 17 (11.2) | 15 (9.0) |  |  | 13 (8.6) | 6 (3.6) |  |
| Moderate | 86 (56.6) | 90 (54.2) |  |  | 96 (63.2) | 82 (49.4) |  |
| High | 49 (32.2) | 61 (36.8) |  |  | 43 (28.3) | 78 (47.0) |  |

Data presented are frequency (percentage). Group difference was evaluated using Wilcoxon-Mann Whitney test.

**Supplementary Table 9. Summary of the data from portable device**

|  | **Baseline** | | |  | **Week 12** | | |  | **Week 12 vs. Baseline** | | | |
| --- | --- | --- | --- | --- | --- | --- | --- | --- | --- | --- | --- | --- |
|  | **CG**  **N = 152** | **PNG**  **N = 166** | **Group Difference *P* value** |  | **CG**  **N = 152** | **PNG**  **N = 166** | **Group Difference *P* value** |  | **CG** | | **PNG** | |
|  |  |  |  |  |  |  |  |  | **Within Group Change** | ***P* value** | **Within Group Change** | ***P* value** |
| Steps | 8367.5 (587.0-21596.0) | 8121.0 (632.0-24271.0) | 0.717 |  | 8468.5 (2423.2-22758.0) | 9716.8 (1100.6-19482.0) | 0.0003 |  | -275.90 (3889.00) | 0.288 | 1114.40 (3022.00) | <0.0001 |
| Calories (Kcal) | 159.5 (9-527) | 161.5 (10-684) | 0.992 |  | 154.2 (50.8-509.8) | 185.4 (61-643) | <0.0001 |  | -4.20 (76.00) | 0.275 | 26.70 (79.20) | <0.0001 |
| Sleep Duration (hour) | 7.17 (2.37-11.17) | 7.04 (2.68-10.73) | 0.470 |  | 7.06 (4.66-11.43) | 7.21 (3.22-10.28) | 0.434 |  | 0.18 (1.52) | 0.207 | 0.22 (1.74) | 0.066 |

Data presented are median (1^st^ quartile, 3^rd^ quartile) at baseline and week 12. Group differences were evaluated using Wilcoxon-Mann Whitney test. Within-group change from baseline were evaluated using Wilcoxon signed ranks test.

**Supplementary Table 10. Association between genotype and baseline blood biomarkers**

| **Gene** | **Biomarker** | **Non-risk genotype** | |  | **Risk genotype** | | ***P* value** |
| --- | --- | --- | --- | --- | --- | --- | --- |
|  |  | **N** | **Value** |  | **N** | **Value** |  |
| BCO1 | Vitamin A | 235 | 1.02 (0.28) |  | 83 | 1.03 (0.29) | 0.852 |
| GC | Vitamin 25OHD3 | 288 | 29.37 (8.71) |  | 30 | 24.93 (7.29) | 0.008 |
| MTHFR | Vitamin B9 | 113 | 10.58 5.42) |  | 205 | 8.89 (4.74) | 0.004 |
|  | Vitamin B12 ^a^ | 113 | 402.04 (135.70-854.63) |  | 205 | 349.83 (107.88-1236.27) | 0.090 |
|  | Homocysteine | 113 | 19.83 (6.92) |  | 205 | 21.48 (8.47) | 0.077 |
| FTO | TG ^a^ | 210 | 1.19 (0.38-6.79) |  | 108 | 1.18 (0.35-4.22) | 0.658 |
|  | TC | 210 | 5.28 (0.95) |  | 108 | 5.19 (0.89) | 0.447 |
|  | HDL | 210 | 1.53 (0.28) |  | 108 | 1.47 (0.25) | 0.036 |
|  | LDL | 210 | 2.94 (0.72) |  | 108 | 2.94 (0.71) | 0.961 |
|  | UA | 210 | 319.81 (79.74) |  | 108 | 357.98 (96.55) | 0.0002 |
| APOE | TG ^a^ | 266 | 1.16 (0.35-6.04) |  | 52 | 1.39 (0.60-6.79) | 0.0004 |
|  | TC | 266 | 5.17 (0.90) |  | 52 | 5.64 (0.99) | 0.0008 |
|  | HDL | 266 | 1.50 (0.27) |  | 52 | 1.55 (0.25) | 0.317 |
|  | LDL | 266 | 2.89 (0.68) |  | 52 | 3.17 (0.82) | 0.011 |
|  | UA | 266 | 332.26 (88.82) |  | 52 | 335.37 (81.53) | 0.816 |
| FADS1 | TG ^a^ | 265 | 1.18 (0.35-6.79) |  | 53 | 1.16 (0.49-5.69) | 0.716 |
|  | TC | 265 | 5.28 (0.96) |  | 53 | 5.08 (0.72) | 0.164 |
|  | HDL | 265 | 1.53 (0.27) |  | 53 | 1.43 (0.22) | 0.022 |
|  | LDL | 265 | 2.95 (0.74) |  | 53 | 2.86 (0.59) | 0.366 |
|  | UA | 265 | 335.30 (89.31) |  | 53 | 320.12 (77.75) | 0.250 |
|  | DHA | 265 | 5.72 (1.10) |  | 53 | 5.81 (1.15) | 0.619 |
|  | EPA ^a^ | 265 | 0.39 (0.18-1.44) |  | 53 | 0.31 (0.19-0.78) | 0.002 |
| TCF7L2 | TG ^a^ | 295 | 1.18 (0.35-6.04) |  | 23 | 1.21 (0.55-6.79) | 0.166 |
|  | TC | 295 | 5.24 (0.93) |  | 23 | 5.29 (0.95) | 0.814 |
|  | HDL | 295 | 1.51 (0.27) |  | 23 | 1.50 (0.31) | 0.835 |
|  | LDL | 295 | 2.94 (0.70) |  | 23 | 2.92 (0.84) | 0.928 |
|  | UA | 295 | 329.85 (85.06) |  | 23 | 370.19 (110.45) | 0.033 |
|  | Glucose ^b^ | 295 | 4.70 (3.70-15.50) |  | 23 | 4.70 (3.60-13.00) | 0.470 |
| CaSR | Calcium | 298 | 1.66 (0.11) |  | 20 | 1.66 (0.10) | 0.970 |
| TMPRSS6 | Iron | 66 | 8.76 (0.83) |  | 252 | 8.64 (0.86) | 0.308 |

Data present are mean (standard deviation) or median (1^st^ quartile, 3^rd^ quartile) for non-normal data.

^a.^ Group difference was evaluated using analysis of variance on log10 of the original data.

^b.^ Group difference was evaluated using Wilcoxon-Mann Whitney test.

DHA, docosahexaenoic acid; EPA, eicosapentaenoic acid; HDL, high-density lipoprotein; LDL, low-density lipoprotein; TC, total cholesterol; TG, triglyceride; UA, uric ascid.

**Supplementary Table 11. Association between genotype and intervention effect on anthropometrics**

| **Gene** | **Anthropometrics (Change from Baseline)** | **CG**  **N = 152** | |  | **PNG**  **N = 166** | |  | ***P* value** |
| --- | --- | --- | --- | --- | --- | --- | --- | --- |
|  |  | **Non-risk Genotype** | **Risk Genotype** |  | **Non-risk Genotype** | **Risk Genotype** |  |  |
| *FTO* | Body weight | -0.97 (2.71) | -1.67 (1.85) |  | -1.82 (2.72) | -2.77 (1.71) |  | 0.663 |
|  | BMI | -0.38 (1.04) | -0.65 (0.71) |  | -0.70 (1.03) | -1.01 (0.59) |  | 0.848 |
|  | Waist circumference | -1.28 (2.31) | -2.41 (1.95) |  | -2.66 (2.44) | -3.29 (2.02) |  | 0.352 |
|  | Body fat percent | -0.33 (1.25) | -0.76 (1.09) |  | -1.11 (1.55) | -1.49 (1.48) |  | 0.876 |
| *APOE* | Body weight | -1.08 (2.45) | -1.82 (2.44) |  | -2.05 (2.57) | -2.54 (1.86) |  | 0.740 |
|  | BMI | -0.43 (0.93) | -0.69 (1.02) |  | -0.78 (0.96) | -0.93 (0.60) |  | 0.711 |
|  | Waist circumference | -1.55 (2.25) | -2.31 (2.16) |  | -2.84 (2.42) | -3.02 (1.72) |  | 0.398 |
|  | Body fat percent | -0.41 (1.20) | -0.81 (1.24) |  | -1.17 (1.55) | -1.56 (1.43) |  | 0.989 |
| *FADS1* | Body weight | -1.01 (2.46) | -2.22 (2.18) |  | -1.98 (2.54) | -2.89 (2.02) |  | 0.676 |
|  | BMI | -0.40 (0.94) | 0.83 (0.89) |  | -0.74 (0.94) | -1.10 (0.75) |  | 0.790 |
|  | Waist circumference | -1.50 (2.25) | -2.58 (2.08) |  | -2.75 (2.34) | -3.46 (2.17) |  | 0.593 |
|  | Body fat percent | -0.39 (1.16) | -0.94 (1.40) |  | -1.18 (1.50) | -1.49 (1.72) |  | 0.550 |
| *TCF7L2* | Body weight | -1.25 (2.44) | -0.66 (2.79) |  | -2.15 (2.50) | -1.82 (2.50) |  | 0.807 |
|  | BMI | -0.50 (0.95) | -0.20 (0.93) |  | -0.81 (0.92) | -0.69 (0.96) |  | 0.665 |
|  | Waist circumference | -1.71 (2.28) | -1.30 (1.83) |  | -2.91 (2.34) | -2.41 (1.92) |  | 0.930 |
|  | Body fat percent | -0.47 (1.19) | -0.65 (1.55) |  | -1.31 (1.57) | -0.65 (0.36) |  | 0.059 |

Data presented are mean (standard deviation) of the change from baseline to week 12. The effects of genotype× study group interaction were evaluated using analysis of variance.

**Supplementary Table 12. Association between genotype and intervention effect on blood biomarkers**

| **Gene** | **Blood Biomarkers (Change from Baseline)** | **CG**  **N = 152** | |  | **PNG**  **N = 166** | |  | ***P* value** |
| --- | --- | --- | --- | --- | --- | --- | --- | --- |
|  |  | **Non-risk Genotype** | **Risk Genotype** |  | **Non-risk Genotype** | **Risk Genotype** |  |  |
| *FTO* | TG ^a^ | -0.09 (-2.81-1.50) | -0.10 (-1.64-0.86) |  | -0.23 (-3.94-2.00) | -0.29 (-1.58-0.34) |  | 0.699 |
|  | TC | -0.06 (0.54) | -0.28 (0.58) |  | -0.27 (0.44) | -0.39 (0.34) |  | 0.391 |
|  | HDL | 0.06 (0.21) | 0.11 (0.27) |  | 0.11 (0.17) | 0.13 (0.11) |  | 0.478 |
|  | LDL | -0.21 (0.47) | -0.36 (0.35) |  | -0.33 (0.39) | -0.43 (0.24) |  | 0.584 |
| *APOE* | TG ^a^ | -0.09 (-1.87-1.50) | -0.16 (-2.81-0.85) |  | -0.23 (-2.73-2.00) | -0.27 (-3.94-0.34) |  | 0.966 |
|  | TC | -0.12 (0.58) | -0.22 (0.45) |  | -0.29 (0.44) | -0.40 (0.24) |  | 0.939 |
|  | HDL | 0.07 (0.24) | 0.10 (0.17) |  | 0.11 (0.16) | 0.15 (0.11) |  | 0.732 |
|  | LDL | -0.25 (0.44) | -0.32 (0.44) |  | -0.34 (0.37) | -0.47 (0.24) |  | 0.645 |
| *FADS1* | TG ^a^ | -0.09 (-2.81-1.50) | -0.13 (-1.87-0.42) |  | -0.23 (-3.94-2.00) | -0.27 (-2.96-0.27) |  | 0.892 |
|  | TC | -0.14 (0.58) | -0.14 (0.50) |  | -0.31 (0.43) | -0.29 (0.34) |  | 0.893 |
|  | HDL | 0.07 (0.24) | 0.11 (0.20) |  | 0.10 (0.15) | 0.18 (0.14) |  | 0.453 |
|  | LDL | -0.25 (0.44) | -0.34 (0.40) |  | -0.35 (0.35) | -0.41 (0.35) |  | 0.769 |
| *TCF7L2* | TG ^a^ | -0.09 (-2.81-1.50) | -0.10 (-1.21-0.22) |  | -0.23 (-2.96-2.00) | -0.36 (-3.94-0.69) |  | 0.416 |
|  | TC | -0.15 (0.57) | -0.07 (0.37) |  | -0.29 (0.40) | -0.46 (0.54) |  | 0.247 |
|  | HDL | 0.08 (0.24) | 0.09 (0.16) |  | 0.12 (0.15) | 0.09 (0.19) |  | 0.591 |
|  | LDL | -0.26 (0.44) | -0.30 (0.34) |  | -0.36 (0.34) | -0.43 (0.49) |  | 0.825 |

Data presented are mean (standard deviation) or median (1^st^ quartile, 3^rd^ quartile) of the change from baseline to week 12.

Unless otherwise stated, the effects of genotype× study group interaction were evaluated using analysis of variance (ANOVA).

a) The effect of genotype× study group interaction were evaluated using ANOVA on log 10 of the original data

**Supplementary Figure 1**

Effect of intervention on anthropometrics stratified by physical activity level. Data were mean (standard deviation). Group differences in the change from baseline value were evaluated using analysis of variance.
